# Multi-modality imaging of complex functional tricuspid regurgitation successfully addressed by a patient-tailored ‘zipping by clipping strategy’ with the K-Clip tricuspid annuloplasty system

**DOI:** 10.1093/ehjcr/ytae374

**Published:** 2024-07-30

**Authors:** Zhi-Nan Lu, Yutong Ke, Ziwei Xi, Guangyuan Song

**Affiliations:** Interventional Center of Valvular Heart Disease, Beijing Anzhen Hospital, Capital Medical University, No. 2 Anzhen Road, Chaoyang District, Beijing 100029, China; Echocardiography Department, Beijing Anzhen Hospital, Capital Medical University, No. 2 Anzhen Road, Chaoyang District, Beijing 100029, China; Interventional Center of Valvular Heart Disease, Beijing Anzhen Hospital, Capital Medical University, No. 2 Anzhen Road, Chaoyang District, Beijing 100029, China; Interventional Center of Valvular Heart Disease, Beijing Anzhen Hospital, Capital Medical University, No. 2 Anzhen Road, Chaoyang District, Beijing 100029, China

**Figure:**
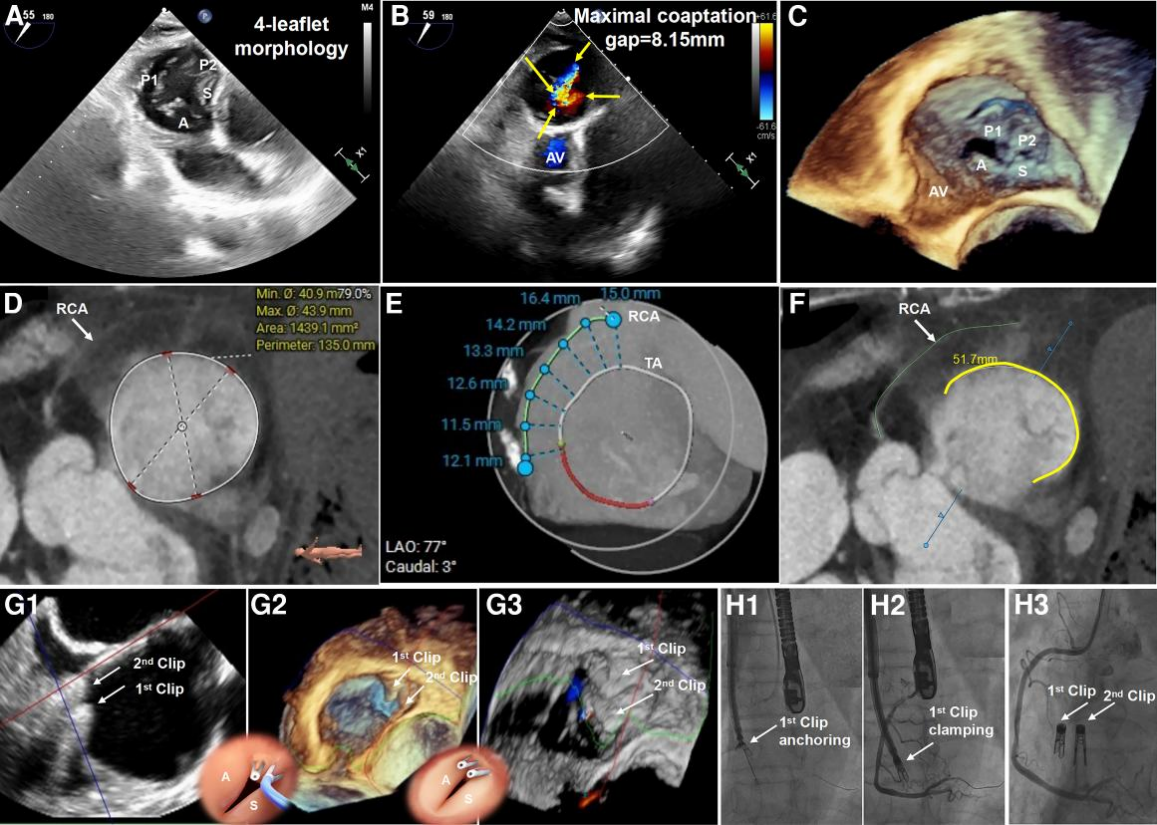


A 78-year-old female with 10-year chronic atrial fibrillation was admitted for refractory peripheral oedema. She was extremely weak but haemodynamically stable. Examination showed a grade 3/6 systolic heart murmur, abdominal swelling with shifting dullness, and severe lower extremity oedema. Electrocardiogram confirmed atrial fibrillation. Transthoracic echocardiography revealed bi-atrial dilatation, torrential tricuspid regurgitation (TR), moderate mitral regurgitation, normal biventricular dimension, and preserved systolic function. Transoesophageal echocardiography demonstrated marked dilated tricuspid annulus leading to a stellate-shaped regurgitant orifice among four leaflets with the maximal coaptation gap of 8.15 mm (*Panel*s *A and B*; Supplementary material online, *Videos S1*–*S3*).

She was evaluated for surgery but declined because of advanced age, frailty, and severe kidney dysfunction with a high society of thoracic surgery score of 15.8% and the European System for Cardiac Operation Risk Evaluation II (EuroSCORE II) of 6.83%. The multi-disciplinary heart team suggested the K-Clip™ transcatheter annuloplasty system (Huihe Medical, China) was a feasible choice. Cardiac computed tomography was arranged to size the tricuspid annulus (*Panel D*) and generate a procedural plan to avoid right coronary artery (RCA) injury (*Panel E*).

Considering the posterior annular length was dilated to 51.7 mm (*Panel F*), a patient-tailored ‘zipping by clipping strategy’ was proposed to maximumly plicate the posterior annulus. The first clip (12 mm) was moved forward to anteroposterior commissure, plicating anterolateral segment of posterior annulus. Then, the second clip (12 mm) was used to clamp the remaining posteroseptal segment, resulting in bicuspidalization and mild residual TR without tricuspid orifice stenosis (mean trans tricuspid valve gradient post-procedure = 2 mmHg) (*Panels G1–G3*; Supplementary material online, *Video S4*). Repeated coronary angiography was performed to confirm unaffected RCA flow during the procedure (*Panels H1–H3*). At 6-month follow-up, the patient’s condition greatly improved with resolved oedema and sustained mild TR.

This is the first case to show based on modality imaging evaluation, the complex functional TR can be successfully addressed by a patient-tailored ‘zipping by clipping strategy’ with the K-Clip system.

(*A*) Significant tricuspid annulus dilatation with extensive huge coaptation gaps among four leaflets was shown in two-dimensional transgastric short-axis view. (*B*) Torrential tricuspid regurgitation with a stellate-shaped orifice and the maximal coaptation gap of 8.15 mm was demonstrated in two-dimensional transgastric short-axis view with colour Doppler. (*C*) Significant tricuspid annulus dilatation with extensive huge coaptation gaps among four leaflets was confirmed in three-dimensional enface view. (*D*) Tricuspid annulus circumstance measured by cardiac computed tomography was dilated to 135 mm. (*E*) The distance between the tricuspid annulus and right coronary artery was measured at multiple points, with a distance of ≥4 mm considered safe for K-Clip implantation without causing right coronary artery injury. (*F*) Posterior tricuspid annulus length was 51.7 mm. (*G1–G3*) Under live two-dimensional and three-dimensional transoesophageal echocardiography guidance with multi-planar reconstruction technique, a novel ‘zipping by clipping’ strategy was employed to close the extensive dilated posterior annulus by segmental clipping the posterior annular tissue from anteroposterior commissure to posteroseptal commissure with two clips (12 mm), resulting mild residual tricuspid regurgitation. (*H1–H3*) Repeated coronary angiographies were performed after the device was anchored and clamped, and again after it was released, to confirm that the right coronary artery flow remained unaffected during the procedure. A, anterior leaflet; AV, aortic valve; P, posterior leaflet; RCA, right coronary artery; RV, right ventricle; S, septal leaflet; TA, tricuspid annulus.

## Supplementary Material

ytae374_Supplementary_Data

## Data Availability

The data underlying this article will be shared on reasonable request to the corresponding author.

## Notes

**Consent:** The authors confirm that written consent for submission and publication of this case report, including images and associated text, has been obtained from the patient in line with Committee on Publication Ethics guidance.

**Funding:** None declared.

