# Supplementary material for: Multi-modality imaging of complex functional tricuspid regurgitation successfully addressed by a patient-tailored ‘zipping by clipping strategy’ with the K-Clip tricuspid annuloplasty system
Source: Eur Heart J Case Rep. 2024 Jul 30;8(8):ytae374. doi: 10.1093/ehjcr/ytae374 (PMC11319875; doi:10.1093/ehjcr/ytae374)
Supplement: ytae374_Supplementary_Data [file ytae374_supplementary_data.zip › Supplemental Materials.docx]

Supplemental Materials

**Video legends**

**Video 1.** Significant tricuspid annulus dilatation with 4-leaflet morphology and extensive huge coaptation gaps was shown in a two-dimensional transgastric short axis view

**Video 2.** Torrential tricuspid regurgitation with a stellate-shaped orifice was shown in two-dimensional transgastric short axis view with color Doppler.

**Video 3.** Significant tricuspid annulus dilatation with 4-leaflet morphology and extensive huge coaptation gaps was confirmed in a three-dimensional view.

**Video 4.** Mild Residual tricuspid regurgitation post-procedure was evaluated by three-dimensional color Doppler with multiplanar reconstruction technique
